# 

**Published:** 1888-09-08

**Authors:** 


**To Residents, Secretaries, and Matrons.**

The Editor will be glad to have the Regulations and each Report as issued by Asylums, Hospitals, Convalescent and Nursing Institutions, as well as those of other Charities, and an early copy in duplicate of all Appeals and Festival Notices, etc., addressed to *The Editor, The Lodge, Porchester Square, London, W.* Any superintendent, secretary, matron, or other chief official who regularly sends such information may be registered, on application to the Publisher, to receive free of cost a cover for binding The Hospital in half-yearly volumes.

**Notice to Advertisers and Subscribers.**

All Advertisements and Business Communications should be addressed to The Publisher, *not to the Editor*, The Hospital, 38, Old Jewry, E.C.
A Scale of Charges will be found in the Advertisement Sheets, page iii.

### FEES.

This is the advice given by our American contemporary the *Medical Record* to medical men on the important subject of fees.

Always make a charge for each service: This gives it a business value in the eyes of the patient.

The charge should always be just and reasonable; then no reduction is necessary.

Insist always on full payment, based, if necessary, upon detailed accounts.

When the patient asks for a reduction of his bill, recall the sacrifice of sleep, of meals, and of comfort in rendering him prompt service.

Never allow sentiment to interfere with business. The "Thank you" is best emphasised by the silvery accent of chinking coin.

The loss of money by sickness only affects one side in every other business. Why should it be different when the doctor is to be paid?

Always charge a fixed fee, and never trust to the patient's generosity nor embarrass him by guessing an amount that would be satisfactory to you. It is very much like firing with a kicking gun at a black cat in the dark.

Render bills at short intervals: be in earnest when you commence to collect them.

### Amusements with Profit for all Ages.

**Rules.**—All answers must be addressed to the Prize Editor, The Hospital, 38, Old Jewry, Cheapside, London, E.C., not later than the *first* post on Thursday next. The full name and address must in all cases be given, but a *nom de plume* may be added for publication. Only one side of the paper must be written on.

### FOR MEN AND WOMEN.

**Rule.**—Competitors can enter for *all* quarterly competitions, but no competitor may take more than *two quarterly prizes* during the year.

### First Quarterly Poetical Competition.

Commenced May 26th, 1888; ended August 25th, 1888.

This quarter a PRIZE of ONE GUINEA will be given to the competitor who gains the highest number of marks for original poems, on any subjects suitable for insertion in this paper. All contributions sent in will be credited according to merit, eighteen marks being the highest score for any one contribution. This competition to alternate weekly with the set acrostic competition.

### Acrostic Competition.

This quarter the original acrostic competition will be discontinued, and A PRIZE of HALF-A-GUINEA will be given to the competitor who gains the highest score for solution of acrostics set. *One mark* only will be given to the competitors who solve all the lights of each acrostic.

Exception will be made in the case of *quotation acrostics*, when *two marks* will be given, *one* for the correct solution of all *lights*, and *one* mark for the *source* of the quotations.

### Results of No. 7 Original Poem.

Dodo (17), Anthem (16), Patience (14) Hesperornis (14).

### A Hymn for use in Hospitals.

Jesu, Lord of Mercy,
Who Thyself didst share
All the pains and sorrows
Which Thy servants bear!
Lo! we come before Thee
Helpless and distrest,
Praying Thee to grant us
Comfort, ease, and rest.

Sin and wayward longings,
Not our Father's will,
Warped our human nature,
And enslaves us still:
He made all things perfect,
Gave them powers for good,
And His own communion
For their daily food.

But when we had fallen
Under sin's dread ban,
By the breach exacting
Penalties from man,
Jesus died to save us,
He our burden bore,
And to plead our ransom
Liveth evermore.

Lord! to thee we hasten
Racked with pain and care,
Thou providest healing
In response to prayer;
Thou alone canst save us,
Thy great power we know,
Trustfully we claim it
For thou lovest so.

By Thy bitter passion
'Neath the tempter's pow'r,
By Thy need of comfort
In Thy trial's hour;
Grant us brave endurance,
Cheer us with Thy love,
Make these wards the portals
To our home above.

—*Dodo.*

### Results of Poetical Competition.

| Names. | Total of Marks. | Names. | Total of Marks. |
|---|---|---|---|
| Dodo | 104 | S. M. W. | 49 |
| Patience | 91 | Anthem | 43 |
| Hesperornis | 84 | Padre | 14 |
| A. M. E. | 78 | Mater | 10 |
| K. M. | 56 | | |

We have forwarded the Prize of One Guinea to "Dodo,"
Rev. W. GOODLIFFE,
7, Park Side,
Cambridge.

### Third Quarterly Acrostic Competition.

| Names. | Total of Marks. | Names. | Total of Marks. |
|---|---|---|---|
| Reldas | 4 | Condorcet | 2 |
| Dodo | 3 | Tinie | 2 |
| Ruffles | 3 | Ellice | 1 |
| Alicia | 3 | K. M. | 1 |
| Lady Clara | 3 | Sycamore | 1 |
| Mater | 2 | Hesperornis | 1 |
| Alice | 2 | Ostrich | 1 |
| Rowdy Corner | 2 | | |

We award the Prize of Half a Guinea to "Reldas,"
Miss ALICE SADLER,
10, Sussex Gardens,
Eastbourne.

### SPECIAL NOTICE.

The Competitions in this column will be suspended until October, when a new scheme will be presented, in which we hope that all our present competitors, and many new ones, will take part.

### THE CHILDREN'S CORNER.

PRIZES.

FOR MOST CORRECT ANSWERS TO PUZZLES.

This Commenced October 1st, 1887.

One Pound a Month.

A PRIZE OF THREE GUINEAS

to the Competitor who shall have sent in the largest number of correct or best answers during the twelve months.

### Puzzles.—Second Week.

No. 5.—Double Acrostic.

Solid and liquid, sour and sweet,
In primals and finals surely meet.

1. The quality of humour or of fun.
2. A great commotion you'll do well to shun.
3. The pride of June, 'mid nature's graces queen.
4. The flower of August, glorious to be seen.
5. That mighty agent, by whose power with ease
We level rocks, or span the stormy seas.

No. 6.—Anagrams on the Names of Celebrated Men.

1. Shame, cry to all. 2. In air, go carve it. 3. Draws deer in lines. 4. Humph! See Jo!

No. 7.—Buried Birds.

1. He travelled either second or third in the train to-day.
2. Muslin, net and tulle are very useful.
3. Go at once to the post, Richard, or it will be too late.

No. 8.—Enigma.

From dust I came, and when I'm turned to dust,
'Tis then in me that men put all their trust.

### Results of Fourth Week Puzzles.

No. 13.—Biographical Acrostic

H orati I
O 'Connel L
M ag I
E mm A
R obin Hoo D

No. 14.—Missing Word Puzzle.

Close on the hounds the hunter came,
To cheer them on the vanished game;
But, stumbling in the rugged dell,
The gallant horse exhausted fell.
The impatient rider strove in vain
To rouse him with the spur and rein;
For the good steed, his labours o'er,
Stretched his stiff limbs to rise no more;
Then, touched with pity and remorse,
He sorrowed o'er the expiring horse.
—"Lady of the Lake."

No. 15. Arithmetical Puzzle.—The bootmaker lost £3 15*s.* and the boots, or £5.

No. 16. Charade —Dandy(e) lion=Dandelion.

| Marks. | August 25th. | Total of Four Weeks. |
|---|---|---|
| A. C. K. | 4 | 16 |
| Alsie | — | 1 |
| Boadicea | 3 | 13 |
| Curly | — | 4 |
| Hercules | — | 2 |
| Poodle | — | 2 |
| Thanet | 3 | 14 |
| Scrooge | 4 | 13 |
| Gem | 3 | 13 |
| Ally | 4 | 15 |
| G. M. M. | 4 | 15 |
| Janette | — | 7 |
| Tynesider | 4 | 14 |
| R. H. | — | 7 |

### Results of Eleventh Monthly Competition.

We divide the prize of One Pound equally between "Ally,"
Master H. SADLER,
Fountain House, Richmond,
Surrey;
and "G. M. M.,"
Miss G. M. MILLS,
Colville House,
Gratwicke Road,
Worthing.

It will be seen by the above scale of marks that A. C. K. has gained the highest score during the past month, but he is only eligible for the yearly prize, having already received one monthly prize since this competition commenced.

### Notice to Correspondents.

*A. M. Smith.*—Puzzles received too late to be credited. They must be sent in weekly.

### Conditions.

I. Every paper sent in must be clearly written, and one side only must be used. All competitors must mark at the head of their papers the number of their competition.

II. Each paper must be signed by the author with his or her real name and address. A *nom de plume* may be added, if the writer does not desire to be referred to by us by his real name. In the case of all prizewinners, however, the real name and address will be published.

**Notice to all Correspondents.**—N.B.—All letters referring to this page, which do not arrive at 38, Old Jewry, Cheapside, London, E.C., by the *first post on Thursdays*, and are not addressed PRIZE EDITOR, will in future be disqualified and disregarded.

No one will be permitted to win the Monthly Prizes twice in any one year, the Annual Prize being offered especially to prevent this.

